# Large-scale genome-wide association studies reveal the genetic causal etiology between ankylosing spondylitis and risk of leukemia and lymphocytic malignancies

**DOI:** 10.3389/fonc.2024.1432664

**Published:** 2024-09-10

**Authors:** Guang Li, Changhu Dong, Yanping Song, Fei Gao

**Affiliations:** ^1^ Xi’an Institute of Hematology, Xi’an Central Hospital Affiliated to Xi’an Jiaotong University, Xi’an, China; ^2^ Department of Hematology, The Second Affiliated Hospital of Shaanxi University of Chinese Medicine, Xianyang, China; ^3^ Department of Hematology, Tianjin Hospital, Tianjin University, Tianjin, China

**Keywords:** ankylosing spondylitis, leukemia, lymphoma, lymphocytic leukemia, myeloid leukemia, multiple myeloma, Mendelian randomization

## Abstract

**Background:**

Evidence from multiple observational studies suggests that ankylosing spondylitis (AS) is associated with leukemia and lymphocytic malignancies. However, the obtained results are inconsistent, and the causal relationship still needs to be determined. In this context, we utilized two-sample Mendelian randomization (MR) to investigate potential causal associations between AS and leukemia and lymphocytic malignancies.

**Methods:**

The analysis was conducted through published genome-wide association studies (GWAS). We obtained genetic data on AS as the exposure and leukemia, including lymphocytic leukemia, myeloid leukemia, and lymphocytic malignancies including lymphoma, multiple myeloma (MM) as the endpoint. The main method to evaluate causality in this analysis was the inverse variance weighting (IVW) technique. Additionally, we employed the weighted mode, weighted median, and MR-Egger regression for supplementary analyses. Finally, heterogeneity tests, sensitivity analyses, and multi-effect analyses are carried out.

**Results:**

In a random-effects IVW analysis, we found that genetic susceptibility to AS was associated with an increased risk of leukemia (OR = 1.002; 95%CI, 1.001–1.003; p = 0.003) and an increased risk of lymphocytic leukemia [OR = 1.001; 95% CI, (1.000–1.002), p = 0.008]. There was no evidence that AS was associated with lymphoma, myeloid leukemia, and MM.

**Conclusion:**

Our research indicates that AS was associated with an elevated risk of leukemia, and further analysis of specific types of leukemia showed that the risk of lymphocytic leukemia was associated with AS. Our findings highlight the importance of active intervention and monitoring to mitigate leukemia, especially lymphocytic leukemia risk in patients with AS.

## 1 Introduction

Leukemia is a group of malignant tumors, which is characterized by immature hematopoietic precursors invading bone marrow and differentiation stagnation at different mature stages. The most convincingly identified causes of leukemia are exposure to ionizing radiation, to some chemicals, and to some anti-cancer drugs (1). The long-term outcome of patients with leukemia still needs to be improved, and new effective therapeutic strategies continue to be an unmet clinical need (2, 3). For the past few years, the role of inflammatory processes in the transformation, survival, and proliferation of leukemias has received increasing attention (4). The pathogenesis of AS, a chronic immune-mediated inflammatory disease, is associated with elevated levels of interleukin-17 and other inflammatory factors (5). Observational studies show that the risks of non-Hodgkin lymphoma (NHL), chronic lymphocytic leukemia (CLL), and MM are increased among elderly patients with AS or rheumatoid arthritis (RA) (6, 7). A nationwide cohort study in Taiwan suggest that patients with AS have an increased risk of developing cancer, especially hematologic malignancies (8). However, there are some studies that deny the relationship between AS and hematologic malignancies risk. The findings of a nationwide Swedish case–control study indicate that patients with AS do not exhibit a significantly elevated risk of lymphoma (9). Additionally, in observational studies, the association between AS and leukemia and lymphocytic malignancies can be easily confounded by various factors such as environmental influences, viral infections, and exposure to ionizing radiation (10). Thus, whether there is a causal relationship between AS and leukemia and lymphocytic malignancies remains uncertain.

In the field of epidemiology, MR analysis, as a natural randomization method, is increasingly used to integrate the summary data of genome-wide association studies (GWAS) (11). It uses exposure-related genetic variation as a proxy index to evaluate the correlation between exposure and outcome and is widely used in the study of causality in disease etiology (12, 13). In addition, MR can effectively eliminate potential bias and confounding factors (such as immune dysfunction, genetic factors, and environmental factors) and ensure the reliability and effectiveness of experimental results (14). In this study, two-sample MR was used to analyze whether there is a causal relationship between AS and the risk of hematological malignancies.

## 2 Materials and methods

### 2.1 Study design

**Figure 1:**
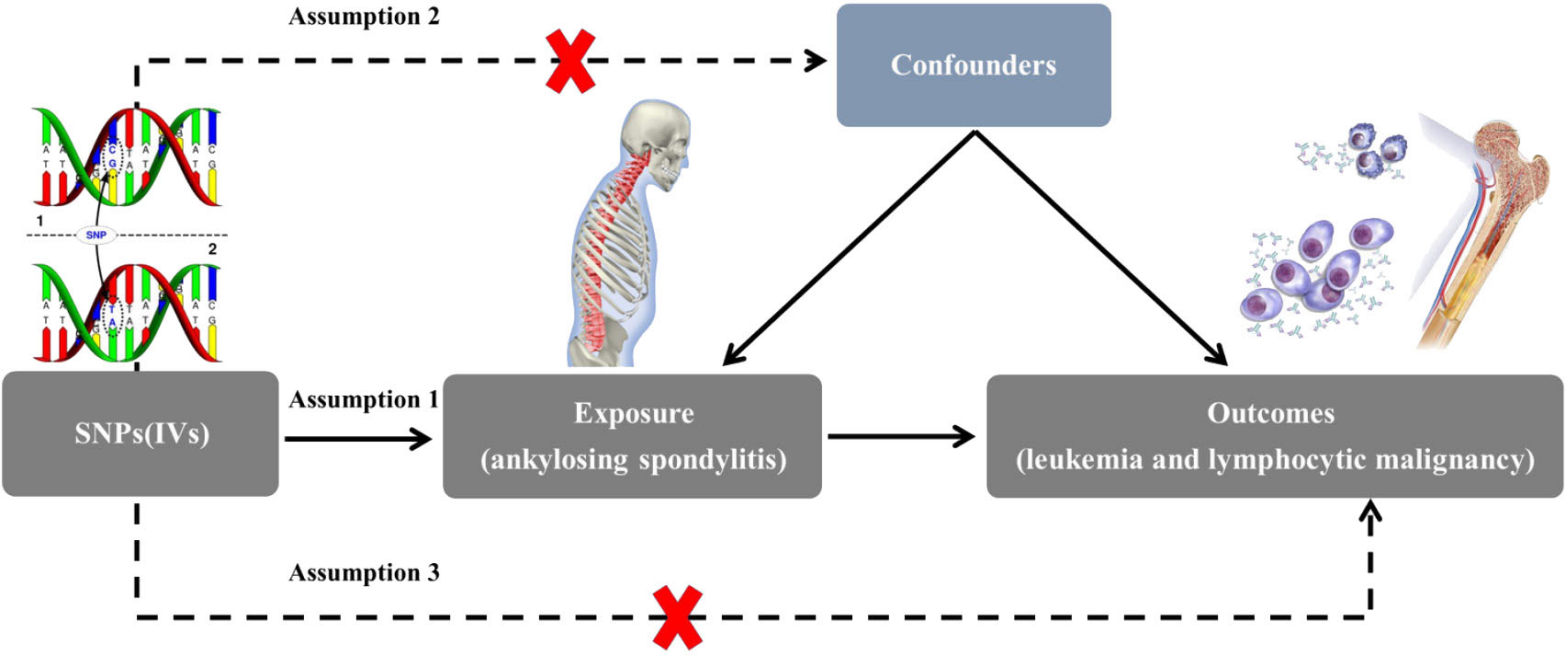
Diagram of the two-sample Mendelian randomization study for the association between AS and risk of leukemia and lymphocytic malignancies. IVs, instrumental variables; SNP, single-nucleotide polymorphisms.

A two-sample MR study was conducted to investigate the causal relationship between AS and leukemia and lymphocytic malignancy. The inverse variance weighting (IVW) approach was primarily used to make causal conclusions about the effect of AS on the development of leukemia and lymphocytic malignancy. Multiple single-nucleotide polymorphisms (SNP) representing human genetic variation were selected as instrumental variables (IVs). The fundamental principle of MR involves the use of genetic variants associated with exposure and outcome as IVs to infer causality. Classical MR analysis employs three key assumptions (**Figure 1**): (1) IVS were directly related to exposure, (2) IVs were independent of any confounding variables; and (3) IVs affect the outcome only through exposure (15, 16).

### 2.2 Data sources

The summary statistics for the exposure and outcome variables were obtained from the IEU Open GWAS Database Project at https://gwas.mrcieu.ac.uk/. The details of the data source and definition are listed in **Table 1**. Our analyses were based on publicly available aggregated GWAS data. Therefore, ethics committee approval was not required.

**Table 1:** Data sources.

Traits	Dataset	Sample size	SNPs	Cases	Controls	Population
Exposure						
AS	ebi-a-GCST005529	22,647	99,962	9,069	1,550	European
Outcomes						
Leukemia	ieu-b-4914	373,276	9,980,879	1,260	372,016	European
lymphoma	ukb-d-C_LYMPHOMA	361,194	10,226,672	1,752	359,442	European
Lymphocytic leukemia	ieu-b-4956	372,776	9,015,063	760	372,016	European
Myeloid leukemia	ieu-b-4958	372,478	8,171,258	462	372,016	European
MM	ieu-b-4957	372,617	8,615,746	601	372,016	European

AS, ankylosing spondylitis; MM, multiple myeloma; Cases, indicates the patient count in this GWAS; Controls, denotes the count of healthy controls included in this GWAS.

### 2.3 IVs selection and evaluation

**Figure 2:**
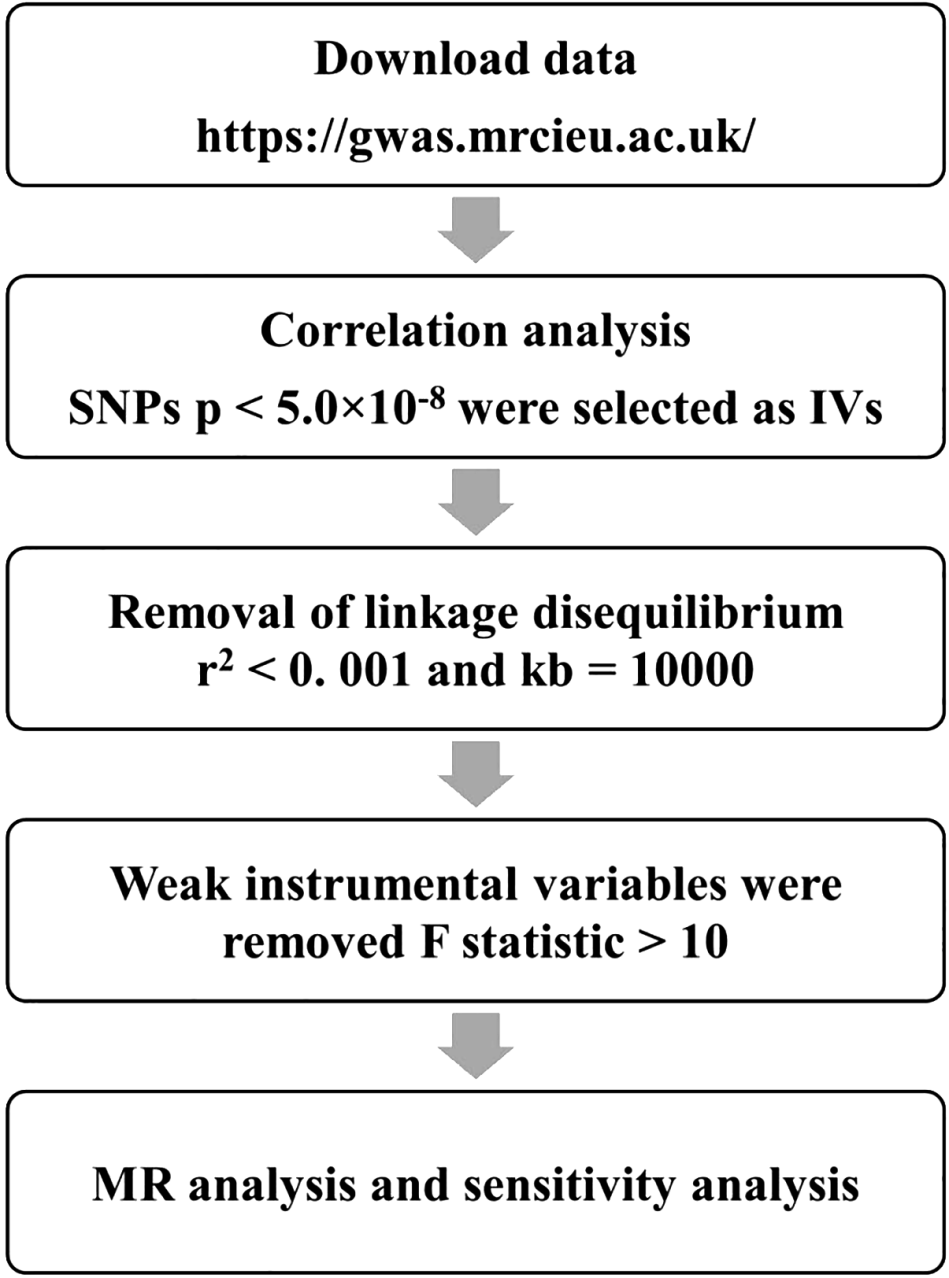
Screening conditions and analysis procedures of IVs in MR analysis.

Initially, significant SNPs associated with the exposure variable were extracted as IVs from the IEU Open GWAS database. SNPs associated with AS at the genome-wide significance threshold p < 5.0×10^−8^ were selected as potential IVs. Second, adjust linkage disequilibrium (LD). LD refers to the non-random association between alleles of different loci, that is, as long as two genes are not completely independent, they will show a certain degree of linkage, so set parameters R^2^ < 0. 001 and KB = 10,000 to ensure the independence between genetic tools. Additionally, to evaluate the potential bias from weak IVs, we calculated the F-statistics for each SNP using the formula F=β^2^/SE^2^ (**Figure 2**). SNPs with an F-statistics exceeding 10 were selected to lessen the likelihood of bias stemming from weak IVs (17).

### 2.4 Mendelian randomization analyses

The “Two Sample MR” packages (version 0.5.7) of the R software (version 4.3.1) were used to perform MR analysis (18). A p-value <0.05 was considered statistically significant. In this study, five Mendelian randomization analysis methods were used to determine the causal relationship between AS and leukemia and lymphocytic malignancy, including inverse variance weighted (IVW) (19), weighted median (WM) (20), MR-Egger (21) analysis method, simple mode method, and weighted mode method. Among them, IVW method thinks that SNPs have no multidirectional existence, and the existence of intercept term is not considered in regression, which is regarded as the main analysis method. To compensate for the limitation of IVW, which assumes all genetic variables to be valid IVs, we employed the weighted median method. This approach allowed us to amalgamate data from multiple genetic variables into a single consistent causal assessment, even if up to 50% of the information originated from potentially invalid IVs (20, 22).

### 2.5 Sensitivity analysis

In this investigation, the MR-Egger regression method was utilized to evaluate the potential pleiotropic effects of all SNPs. MR-Egger can evaluate pleiotropy, and the existence of intercept term is considered in regression. If the intercept term is very close to 0, the MR-Egger regression model is very close to IVW. On the contrary, it means that there may be horizontal pleiotropy between these IVs (23). WM is a supplement to MR-Egger regression method. If at least 50% of the weights come from valid IV, the weighted median will provide a consistent estimate. The Cochran’s Q test is used to detect heterogeneity; when p < 0.05, there is heterogeneity, and random effect model is used (24). Instead, use the fixed effect model. Use the “leave one out method” to evaluate the influence of each SNP on the overall results and generated a forest plot to illustrate the results.

## 3 Results

### 3.1 Characteristics of the selected SNPs

We identified 26 robust SNPs as IVs for AS based on established quality control criteria. The F-statistics of the vast majority of these SNPs were above the threshold of 10, which indicated that they strongly represent AS in the MR analysis. The detailed characteristics of these IVs are displayed in **Supplementary Table S1**. Therefore, these IVs were robust and feasible for assessing the causal association between AS and leukemia and lymphocytic malignancies.

### 3.2 MR analysis of AS and leukemia and lymphocytic malignancy

**Figure 3:**
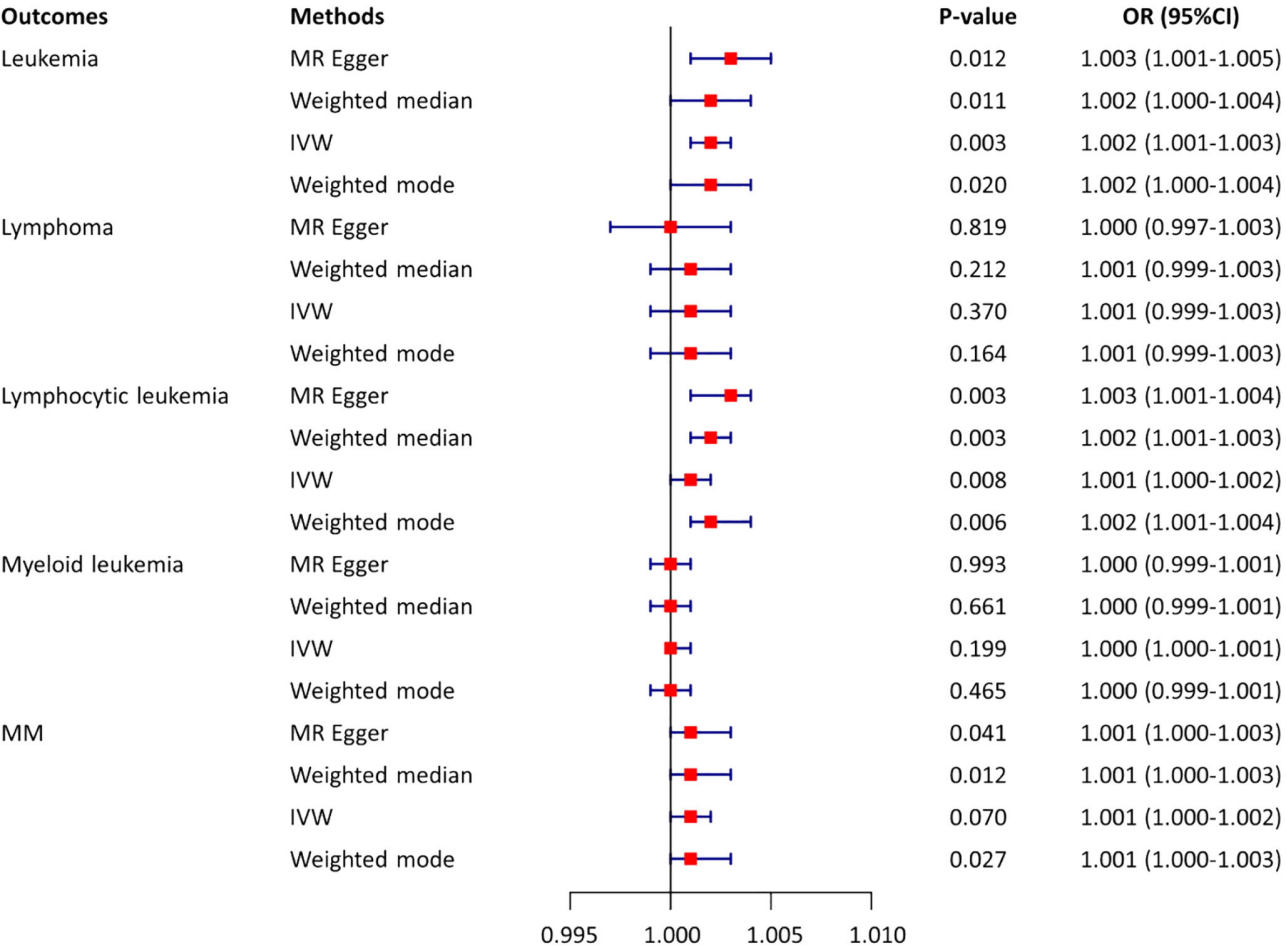
Estimates from Mendelian randomization analysis of AS and risk of leukemia and lymphocytic malignancies. OR, odd ratio; MM, multiple myeloma; CI, confidence interval; IVW, inverse variance weighting.

**Figure 4:**
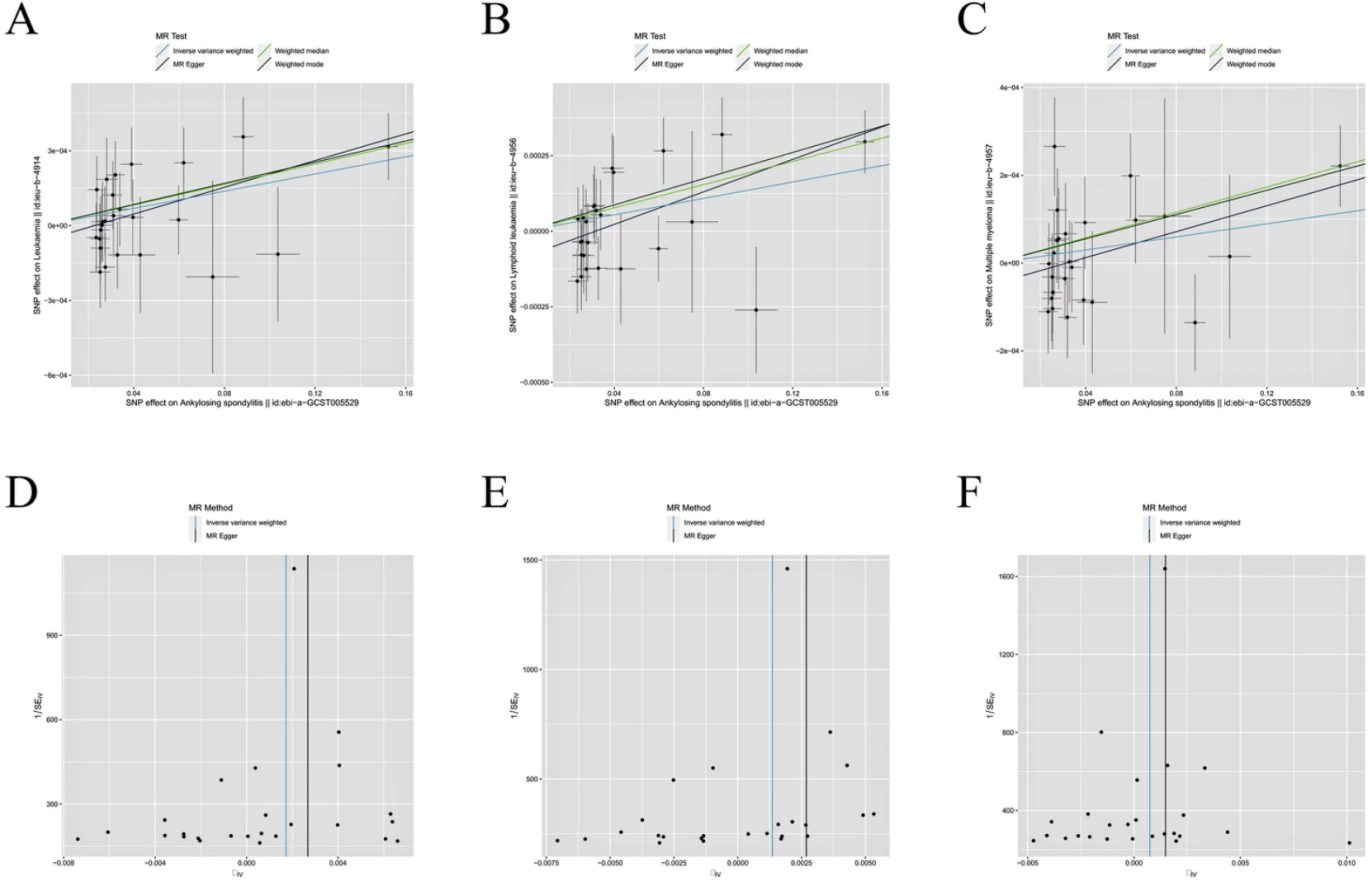
Scatter plot and funnel plot incorporating all IVs. **(A, D)** AS and leukemia; **(B, E)** AS and lymphocytic leukemia; **(C, F)** AS and multiple myeloma.

The causal relationship between AS and leukemia and lymphocytic malignancies was analyzed using MR analysis. This allowed us to test a total of five causality pairs, of which two were statistically significant. In a random-effects IVW analysis, we found that genetic susceptibility to AS was associated with an increased risk of leukemia (OR = 1.002; 95%CI, 1.001–1.003, p = 0.003) (**Figures 3**, **4A**) and an increased risk of lymphocytic leukemia [OR = 1.001; 95% CI (1.000–1.002); p = 0.008) (**Figures 3**, **4B**). The MR-Egger regression and weighted median analyses showed that the IVW association pattern remained directionally consistent inmost statistical models, demonstrating the robustness of the inferred causal relationships between AS and total leukemia and lymphocytic leukemia (**Figure 3**). The risk of MM in genetically predicted AS patients had an increasing trend with marginal statistical effect in the IVW analysis (OR = 1.001; 95% CI, 1.000–1.002; p = 0.07) (**Figures 3**, **4C**). Otherwise, there was no evidence of a causal relationship between AS and lymphoma (OR = 1.001; 95% CI, 0.999–1.003; p = 0.370) and AS and myeloid leukemia (OR = 1.000; 95% CI, 1.000–1.001; p = 0.199) in the IVW analysis results (**Figure 3**).

### 3.3 Sensitivity analysis of Mendelian randomization

In the Cochran’s Q-test, p-values of Q-statistics in the AS and lymphoma analyses were lower than 0.05, indicating IVs heterogeneity (**Table 2**, **Supplementary Figure S1**) and justifying the use of a random effects model in these cases. The remaining IVW analyses showed no heterogeneity in IVs (**Figures 4D-F**), indicating that random effects or fixed effects models can be used in these cases. Concurrently, we note that no MR-Egger regression intercepts deviated from zero (**Supplementary Figure S2**), and no evidence for horizontal pleiotropy in the IVs associated with both AS and leukemia and lymphocytic malignancies (all intercept p > 0.05) (**Table 2**). Finally, the leave-one-out analysis confirmed that no causal associations were driven by a specific IV (**Supplementary Figure S3**).

**Table 2:** Multiplicity and heterogeneity tests for the associations between AS with leukemia and lymphocytic malignancy.

Outcomes	Heterogeneity test		Pleiotropy test		
	Q-pval	Q	Egger_intercept	SE	p
Leukemia	0.7545	18.9538	−6.08e−05	5.07e−05	0.242
Lymphoma	0.0438	36.9986	2.79e−05	7.81e−05	0.725
Lymphocytic leukemia	0.1786	30.1871	−8.49e−05	4.15e−05	0.053
Myeloid leukemia	0.5864	21.8801	2.93e−05	3.08e−05	0.351
MM	0.4167	24.8000	−4.68e−05	3.51e−05	0.196

Q, heterogeneity statistic Q; SE, standard error; MM, multiple myeloma.

## 4 Discussion

AS is a chronic inflammatory autoimmune disease that primarily affects the axial joints but can also involve peripheral joints and various organs. The onset of AS is related to genetic factors, immune responses, and bone formation pathways, but the exact mechanism is not yet clear. Epidemiological surveys show that the prevalence of the disease is approximately 0.55% of the Caucasian population and 0.26% in Chinese, and men are more likely to develop the disease than women, with faster progression, more severe symptoms, and worse outcomes (25). A variety of studies have shown that the levels of inflammatory factors in AS patients are significantly increased, and inflammatory factors are also independent risk factors for AS disease activity (26). Various inflammatory cytokines are elevated in leukemia, and MDS and contribute to dysplastic differentiation. Inflammatory pathways mediated by interleukin (IL) 1b, IL-6, IL-1RAP, IL-8, and others lead to growth of aberrant leukemia and MDS stem and progenitors while inhibiting healthy hematopoiesis (27). Single-cell multi-omic analysis of hematopoietic stem/progenitor cells (HSPCs) revealed chronic inflammation as a driver of TP53-mutant leukemic evolution (28). Recent studies have reported the relationship between inflammatory environment and AML and confirmed this relationship through immunophenotyping of leukemia microenvironment, cytokine profile of AML patients’ plasma, and the imbalance expression of inflammation-related genes found through a large number of RNA gene expression studies (29–32). A variety of endogenous and exogenous factors affect the transformation and progression of leukemia. Intrinsic factors include genetic alterations in cellular pathways that lead to activation of inflammatory pathways such as NF-κB (33, 34). Exogenous components include inflammatory pathways activated by the bone marrow microenvironment, including chemokines, cytokines, and adhesion molecules (4). The above results suggest that AS patients are in a chronic inflammatory state for a long time, and the levels of inflammatory factors in the body are increased. The activation of inflammatory signaling pathways and the increase in inflammatory factors are related to the occurrence and development of leukemia and other malignant hematological diseases, to some extent, revealing the relationship between AS and hematological malignancies.

Hematological malignancies such as leukemia, lymphoma, and MM, are a well-recognized global public health concern, with significant impacts on human health and quality of life (35). AS may increase the risk for the development of malignancies, predominantly lymphoproliferative disorders (36), and sustained inflammatory activity seems to be the primary risk factor for malignancies in AS. As mentioned in *Introduction*, several studies have indicated that the AS may play a role in the onset and progression of leukemia and lymphocytic malignancies, and the reason may be related to inflammation aids proliferation and survival of malignant cells proliferation and survival, stimulating angiogenesis and metastasis, and destroying adaptive immunity (37). A meta-analysis of the malignant tumor risk of patients with AS showed that AS is associated with a 14% increase in the overall risk of malignant tumor. The risk of malignant tumors of the digestive system, MM, and lymphomas is also significantly increased. In the subgroup analysis, patients from Asia have the highest risk of malignant tumor (38). A retrospective clinical study from Peking University First Hospital in China suggested that malignancy is not uncommon in AS patients. Bladder cancer was the most common, followed by hematological malignancies (39). Similarly, retrospective cohort studies from Korea and the United States have both suggested an increased risk of solid tumors in AS patients (40, 41). At the same time, there are many case reports of AS complicated with leukemia and lymphoma (42, 43). In addition, various rheumatic diseases can arise as a consequence of oncology treatment. The interplay between hematological malignancies and immune-mediated inflammatory diseases (IMIDs) such as AS is complex, and the resulting tumor-associated rheumatic diseases represent a rare and intricate group of conditions that occur in the context of malignant tumors (44, 45). Based on the above, we can find that IMIDs, especially AS, may be associated with a high risk of malignant tumors, especially hematological malignancies, such as leukemia and lymphoid tumors. At the same time, chemotherapy or immunotherapy for malignant tumors may induce IMIDs or aggravate the original IMIDs, which are mutually causal (46–48). However, as mentioned in Introduction, observational studies in some countries or regions suggested that AS is not associated with the occurrence of malignant tumors. Up to now, it is not clear whether there is a causal relationship between these two diseases and in what direction these two disease entities were related to each other.

To our knowledge, this is the first study to systematically explore the potential causal relationship between AS and leukemia and lymphocytic malignancies risk using the MR Approaches. MR has been proposed as an alternative method for making causal inference, with the main advantage that the method can often be applied to existing cross-sectional study datasets. Therefore, results can be obtained faster and cheaper in MR studies than in randomized clinical trials (RCTs) (49). MR is an ideal method to explore causal relationships avoiding reverse causality and potential confounding factors (50). Thus, we conducted a two-sample MR study to comprehensively reveal the potential genetic causal effect of AS on leukemia and lymphocytic malignancies, rendering the conclusions more convincing.

Our results suggest that genetic predisposition to AS is associated with an increased risk of total leukemia and lymphocytic leukemia. The risk of MM in genetically predicted AS patients had an increasing trend with marginal statistical effect. However, no MR evidence supports potential causality between genetic predisposition to AS and the risk of lymphoma and myeloid leukemia. Contrary to our findings, in a North American cohort of more than 11 million participants, older patients with AS were associated with an increased risk of hematologic malignancies (primarily NHL, CLL, and MM) (6). Current data from the US Surveillance, Epidemiology, and End Results (SEER) Program reported that compared to men aged 45, the risk of developing NHL in men aged 70 increases by a factor of 7, while the incidence of MM even rises by a factor of 11 (51). Age may serve as one of the confounding variables in this observational study. In addition, hematologic malignancies may develop only after prolonged inflammation, and therefore, the increased risk may be seen only in the elderly. According to the results obtained from our MR analysis, it would be reasonable from a clinical perspective to regularly monitor individuals diagnosed with AS for any potential occurrence of MM and lymphocytic leukemia. Our findings do not provide evidence for a correlation between genetic predisposition to AS and the susceptibility to myeloid leukemia. However, numerous studies in the past decade have linked dysregulation of inflammatory and immune response pathways to the development of myeloid leukemia (52, 53). Possible reasons for this phenomenon in AS may be associated with elevated levels of cytokines and growth factors, which can potentially induce deoxyribonucleic acid (DNA) damage and chromosomal instability, thereby increasing the susceptibility of affected cells to malignant alterations (54). The increased production of myeloid cells often occurs during inflammatory responses. It is crucial to promptly suppress inflammation when normal tissue homeostasis is restored, in order to prevent the development of chronic inflammation, which is associated with premature aging phenotypes and the acquisition of myeloid neoplasms (55, 56). Therefore, the risk association between chronic inflammatory diseases such as AS and myeloid leukemia warrants further attention, necessitating the design of clinical trials and case studies for confirmation. Another retrospective cohort study from Western Australian compared the risk of cancer and subsequent mortality in patients with AS compared with a non-AS population group. This study demonstrates that there is no association between AS and increased risk of cancer diagnosis, while AS is associated with elevated 5-year mortality following a cancer diagnosis (57). The inconsistency in the results of these observational studies highlights the need for MR analysis.

Our research has the following advantages. First, we made MR analysis of the risk of AS and hematological malignancies for the first time. The results of this type of research can avoid being influenced by mixed factors and reverse the causal relationship. Second, we use the latest GWAS data set to ensure that there is no overlap between the exposure and the results, which improves the reliability of the research results. Third, according to the set screening conditions, the sensitive IVs are selected. Fourth, various analytical methods are used, and similar results are always obtained. In addition, through sensitivity analysis, the robustness of the results is ensured.

However, this study also has some limitations. First of all, in order to minimize the statistical bias caused by population stratification, the population that we studied is entirely European, so it is uncertain whether this result can be extrapolated to other races. Second, due to the limited published GWAS data, it is difficult to conduct stratified analysis according to factors such as age and gender, so further subgroup analysis cannot be conducted. Third, the subtypes of diseases (such as lymphoma including diffuse large B-cell lymphoma, mantle cell lymphoma, and follicular lymphoma) were not analyzed by MR, and the correlation between AS, and it was not evaluated separately. We look forward to a comprehensive study in the future and can further study this potential relationship.

## 5 Conclusion

Our research indicates that AS was associated with an elevated risk of leukemia, and further analysis of specific types of leukemia showed that the risk of lymphocytic leukemia was associated with AS. Our findings highlight the importance of active intervention and monitoring to mitigate leukemia, especially lymphocytic leukemia risk in patients with AS.

## Data Availability

The original contributions presented in the study are included in the article/**Supplementary Material**. Further inquiries can be directed to the corresponding author.
