# Supplementary material for: Large-scale genome-wide association studies reveal the genetic causal etiology between ankylosing spondylitis and risk of leukemia and lymphocytic malignancies
Source: Front Oncol. 2024 Sep 10;14:1432664. doi: 10.3389/fonc.2024.1432664 (PMC11419960; doi:10.3389/fonc.2024.1432664)
Supplement: Supplementary file 1 [file DataSheet1.docx]

Supplementary Material

**Supplementary Figure 1.** Funnel plot for IVW and MR-Egger method. (A) AS and leukemia; (B) AS and lymphoma; (C) AS and lymphocytic leukemia; (D) AS and myeloid leukemia; (E) AS and multiple myeloma.

**
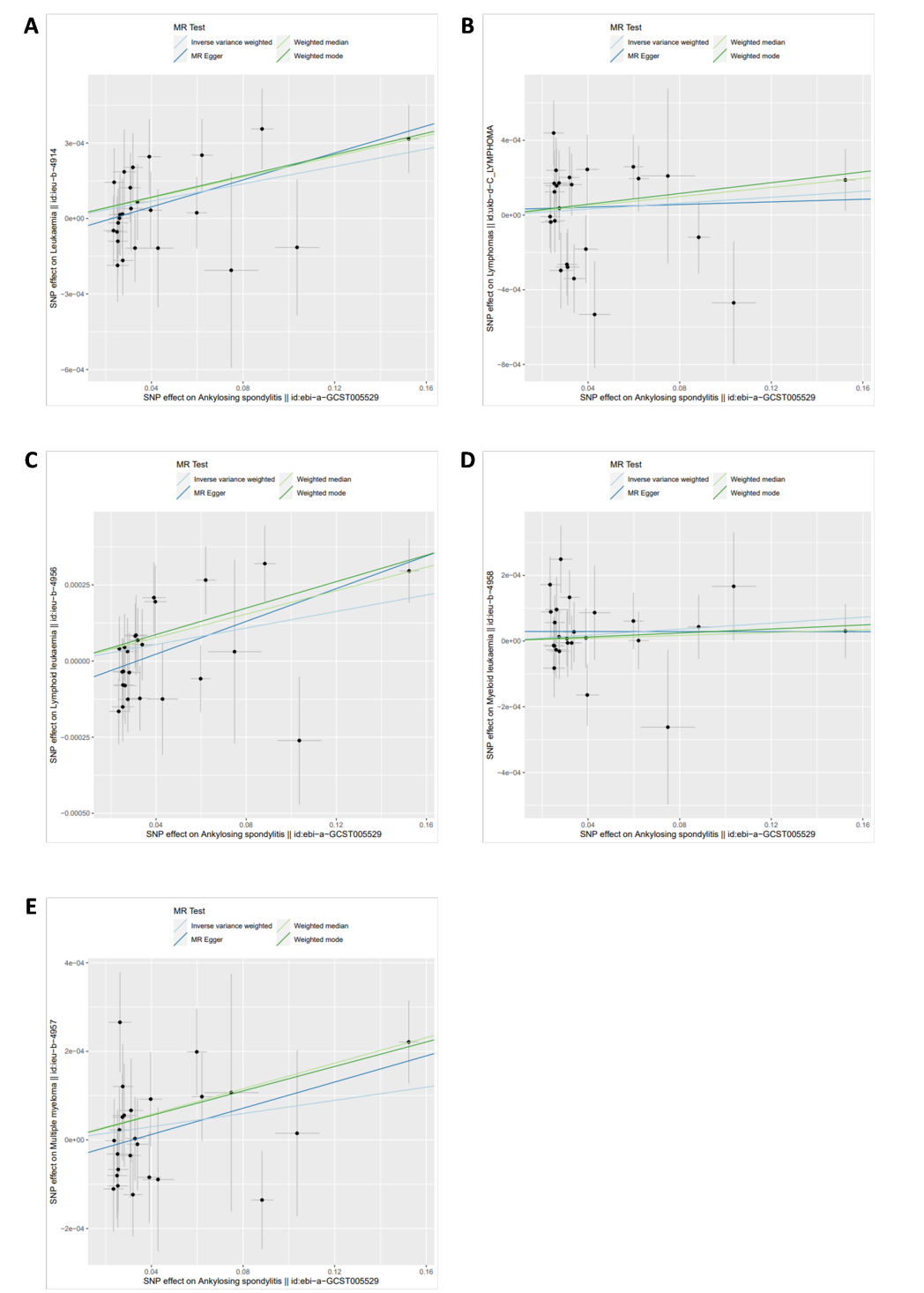
**

**Supplementary Figure2.** Scatter plot using all IVs. (A) AS and leukemia; (B) AS and lymphoma; (C) AS and lymphocytic leukemia; (D) AS and myeloid leukemia; (E) AS and multiple myeloma.

**
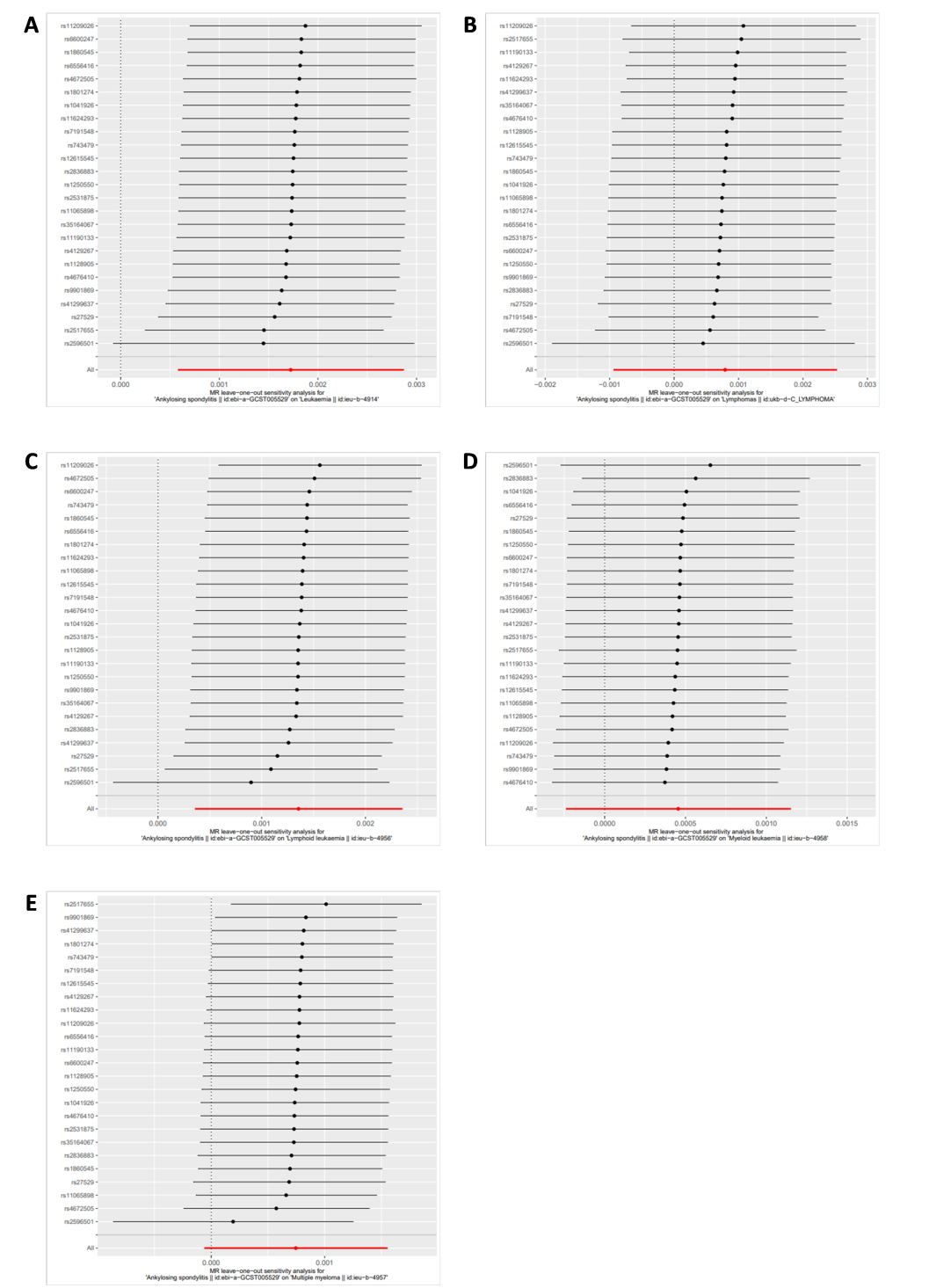
**

**Supplementary Figure 3**. Leave-one-out sensitivity analysis. (A) AS and leukemia; (B) AS and lymphoma; (C) AS and lymphocytic leukemia; (D) AS and myeloid leukemia; (E) AS and multiple myeloma.
